# Pathological assessment of morcellated tissue after ERBT: insights from a two-round Delphi survey

**DOI:** 10.1007/s00345-026-06451-9

**Published:** 2026-05-08

**Authors:** Yossi Ventura, Andrey Morozov, Ronald Chan, Marcelo Combat Faria Tavares, Jeremy Yuen-Chun Teoh, Eva Compérat, Liang Cheng, Eddie Fridman, Julia Lerner, Ezra Baraban, Max Yakimov, Konstantin Lokshin, David Lifshitz, Shay Golan, Vineet Gauhar, Thomas R. W. Herrmann, Shahrokh Shariat, Dmitry Enikeev

**Affiliations:** 1 Department of Urology, Rabin Medical Center, Hasharon Hospital, Petah Tikva, Israel; 2 Institute for Urology and Reproductive Health, Sechenov University, Moscow, Russia; 3 Department of Anatomical and Cellular Pathology, Prince of Wales Hospital, The Chinese University of Hong Kong, Hong Kong, China; 4 Pathologic Anatomy Laboratory, Federal University of Minas Gerais (UFMG), Brazilian Company of Hospital Services, Clinical Hospital, Belo Horizonte, MG Brazil; 5 Department of Surgery, S. H. Ho Urology Centre, The Chinese University of Hong Kong, Hong Kong, China; 6 Department of Pathology, Tenon Hospital, Sorbonne University, Paris, France; 7 Department of Pathology, Medical University of Vienna, Vienna, Austria; 8 Department of Pathology and Laboratory Medicine, Department of Surgery (Urology), Brown University Warren Alpert Medical School, the Legorreta Cancer Center at Brown University, Brown University Health, Providence, RI USA; 9 Department of Diagnostic Pathology, Sheba Medical Center, Ramat Gan, Israel; 10 Gray Faculty of Medical and Health Sciences, Tel Aviv University, Chaim Levanon St 55, Tel Aviv-Yafo, 6997801 Israel; 11 Institute for Clinical Morphology and Digital Pathology, Sechenov University, Moscow, Russia; 12 Department of Pathology, Johns Hopkins University School of Medicine, Baltimore, MD USA; 13 Pathology Department, Rabin Medical Center, Petah Tikva, Israel; 14 Kent and Canterbury Hospital, East Kent Hospitals University Foundation, Canterbury, UK; 15 Ng Teng Fong General Hospital (NUHS), Singapore, Singapore; 16 Asian Institute of nephrourology (AINU), Chennai, India; 17 Department of Urology, Spital Thurgau AG, Kantonspital Frauenfeld, Frauenfeld, Switzerland; 18 Division of Urology, Department of Surgical Sciences, Stellenbosch University, Western Cape, Stellenbosch, South Africa; 19 Hannover Medical School, Hannover, Germany; 20 Department of Urology, Comprehensive Cancer Center, Medical University of Vienna, Vienna, Austria; 21 Hourani Center for Applied Scientific Research, Al-Ahliyya Amman University, Amman, Jordan; 22 Department of Urology, University of Texas Southwestern Medical Center, Dallas, TX USA; 23 Department of Urology, Weill Cornell Medical College, New York, NY USA

**Keywords:** Morcellation, Bladder tumour, Delphi survey, Piece-meal, ERBT, TURBT, Pathology

## Abstract

**Purpose:**

To evaluate the pathological adequacy of morcellated ERBT specimens using a two-round Delphi methodology and to establish expert consensus among leading pathologists worldwide.

**Methods:**

Core pathological parameters relevant for ERBT specimens were predefined, and representative high-quality digital slides of morcellated ERBT specimens were selected. These images were reviewed by an international panel of expert uropathologists with extensive experience in bladder cancer. A two-round Delphi survey was conducted, in which panelists rated the importance of predefined pathological parameters on a 5-point Likert scale. The items on which a consensus was not reached during the first round, were reviewed and rephrased for the second round involving the same responders. Consensus was defined as > 75% agreement among participants.

**Results:**

10 responders took part in the survey. During the initial Delphi round, agreement was achieved on six points, covering aspects such as tumor grading and staging (including lympho-vascular invasion, perineural invasion, detrusor muscle presence, grading adequacy, and histological classification). A broader statement also reached consensus, indicating that morcellation following ERBT provides specimen of appropriate quality. After revising the statements, the second round resulted in consensus on three more items: morcellated specimens allow for precise staging, evaluation of detrusor muscle invasion, and identification of carcinoma in situ.

**Conclusion:**

Tissue acquired after ERBT through morcellation permits dependable pathological evaluation, encompassing grading, staging, and identification of critical prognostic indicators such as carcinoma in situ, detrusor muscle, lymph vascular and perineural invasion. Morcellation is not perceived to compromise pathological evaluation based on expert consensus.

## Introduction

En bloc resection of bladder tumor (ERBT) was first introduced about forty years ago and has since been refined with technological advancements, gaining international recognition. Research suggests that this technique may offer several advantages, such as higher detection rates of the detrusor muscle, fewer residual tumors, improved surgical margins, a reduction in adverse events like bladder perforation, and lower recurrence rates. Some authors hypothesize that it can reduce circulating tumor cells [1–6]. The use of new laser technologies has enhanced safety, precision, and the quality of tissue samples by minimizing cautery-related damage [7, 8]. However, even though many clinical trials have been conducted, there is still not enough strong evidence to prove that ERBT is superior [9, 10] with most researchers opining that 3 cm is the upper limit for the procedure. The possibility of inadequate pathological reporting of tumor specimens removed piece meal is presumed to defeat the purpose of en bloc advantages in single specimen reporting.

Several methods have been developed to perform ERBT in larger lesions. In 2018, morcellation was proposed for bladder lesion removal [11]. The most common approach is a two-step technique: first, resect the exophytic portion; second, excise the base. After separating the tumor, morcellation extracts the specimen for analysis [12]. Alternatively, the tumor, its base, and surrounding tissue can be removed together before morcellation. This method reduces tissue fragmentation compared to piecemeal resection and enables faster, more efficient specimen retrieval. These advantages help simplify pathological processing.

Morcellation is not commonly used, as some believe it may affect tissue structure. Since bladder cancer treatment depends on histologic features, accurate pathologic assessment is crucial. In prostate enucleation, morcellation is as good as a standard resection for cancer detection, with most structural damage caused by the laser. Morcellation can remove large tumors efficiently and may lower recurrence risk by minimizing tumor cell spread, though this benefit remains theoretical and robust data for non-muscle invasive bladder tumors are lacking [11]. 

If proven beneficial, morcellation could become standard practice to improve staging and decision-making. Standardization of pathology reporting is one of the key points for reliable and accurate diagnosis, it requires collaboration between pathologists and urologists. Current standards only in brief mention ERBT and don’t provide any data about morcellation influence on specimens quality [12, 13]. This gap may be addressed by a consensus among pathologists based on interobserver evaluations.

The current article aimed to evaluate the pathological adequacy of morcellated ERBT specimens using a two-round Delphi methodology and to establish expert consensus among leading pathologists worldwide.

## Materials and methods

### Development phase

The methodology was created according to DELPHISTAR checklist and using insights from expert opinions and a recent literature review focused on the ERBT technique and morcellation. To start, we reviewed existing literature about the use of morcellation for bladder tumor removal, highlighting both its advantages and potential challenges [11]. We then held a focus group discussion on key issues of tissue integrity, involving five urologist experts with experience in transurethral resection of bladder tumor (TURBT) and ERBT (at least 50 procedures each) and two pathologist specialists who regularly work with bladder tumors. Each expert has worked in their field for over ten years. Following consensus in these discussions, we developed a 15-item, 5-point Likert questionnaire designed to assess specimen quality after ERBT and morcellation; this served as the initial Delphi survey. Next, we selected pathology specimens from ERBT patients whose tumors measured at least 3× 3 cm on preoperative axial imaging and underwent morcellation. All specimens were collected by a single urologic surgeon with significant experience in laser ERBT and morcellation (> 100 cases). The slides created from these specimens were converted into 32 full-slide digital images, which were sent to pathologists. We chose slides without obvious tissue processing artefacts that may interfere pathological assessment, and not more than 2 slides from each patient to provide diverse kinds of tumors (different grade, stage, etc.) Invited panel of responders was chosen from the expert pathologists with at least 5 years of experience specializing in bladder tumors and working at internationally recognized and leading academic center from various countries, including Europe, the USA, Asia, and Israel. The pathologists were not presented with slides after TURBT, because they already had an extensive experience with such specimens. Thus this is not a head-to-head comparative study with matched specimens. Of the 15 pathologists invited, 10 agreed to participate and took part in both rounds of the survey.

### Delphi process

The Delphi process involved two rounds of structured surveys to gather and refine expert opinions systematically. In the first round, an initial survey was developed based on a literature review and insights from focus group discussions. The items on which a consensus was not reached were reviewed and rephrased for the second round. The second round surveyed the same group of experts to further elaborate and validate the findings from the first round. The process aimed to achieve a high level of consensus on identified issues, with a total acceptance rate of > 75%. The final consensus statement was prepared to address the identified issues and provide direction for future investigations into the pathology examination of large bladder tumors extracted employing ERBT and morcellation.

## Results

### First round of Delphi survey

**Fig. 1:**
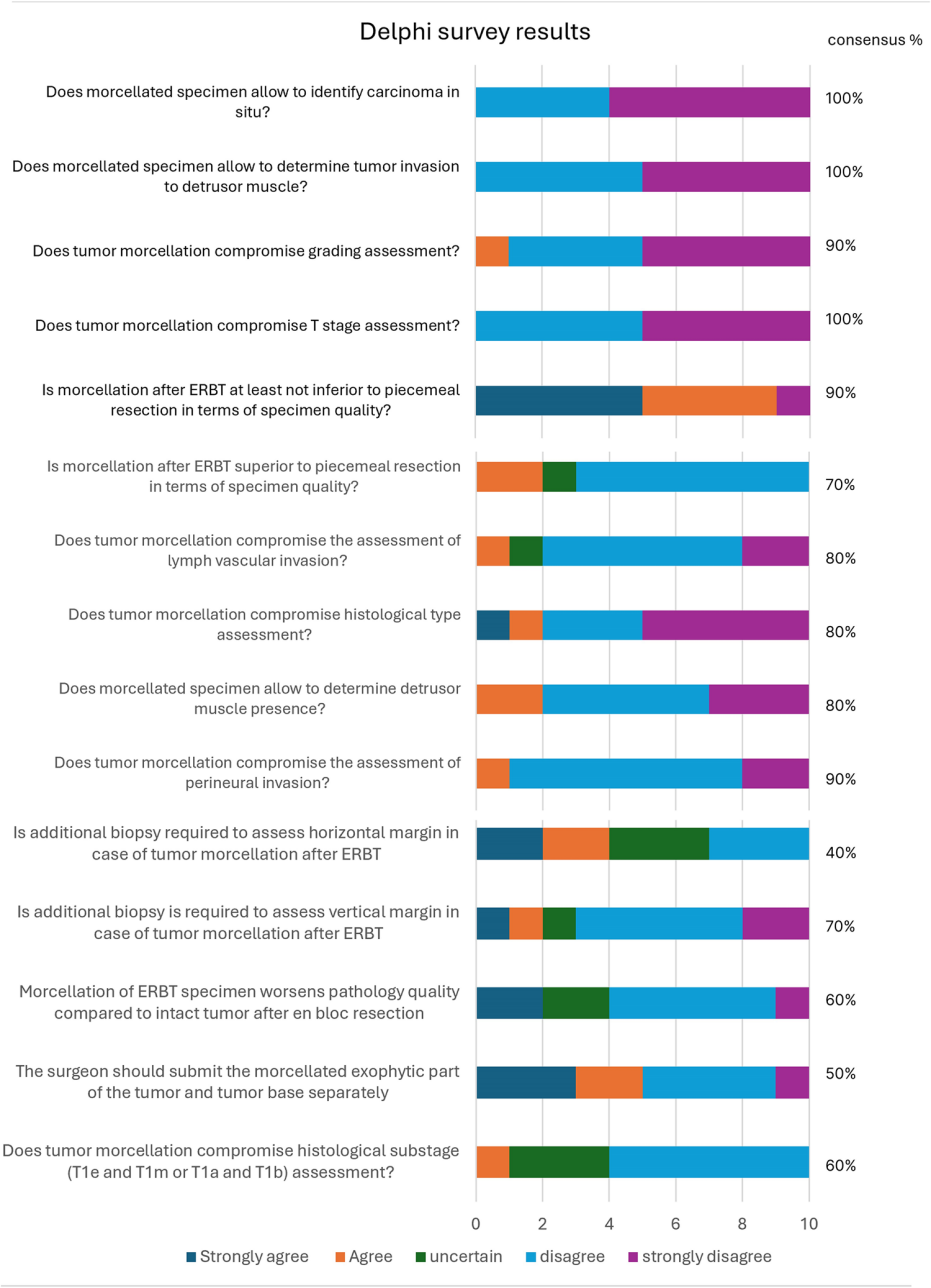
Results of the Delphi survey

The initial survey for the ERBT and Morcellation Consensus Study gathered responses from experts from 8 countries with varying years of experience: 10 of 15 invited responded using the 15-item 5-point Likert questionnaire (supplementary 1). The answers were aggregated and analyzed to identify cohesive quality parameters of ERBT and morcellation. Consensus was achieved for 6 items. Three items reached 90% agreement for the following statements: morcellation after ERBT does not compromise overall specimen quality and assessment of perineural invasion; and morcellation does not impact grading. Three more items reached a consensus among 80% of respondents: morcellation does not compromise lymph-vascular invasion evaluation, morcellation allows detection of detrusor muscle, and morcellation does not compromise histological typing (Fig. 1).

### Second round of Delphi survey

In the second round of the survey with the same responders, 3 essential items reached a 100% consensus: morcellation allows for proper staging, morcellated tissue enables assessment of invasion into the detrusor muscle, and morcellation allows for identifying carcinoma in situ (CIS) (Fig. 1). No consensus was reached on two additional statements addressing the specimen quality, but 70% of respondents agreed that there was no need for a further biopsy to assess vertical margins, and morcellation after ERBT was superior to piecemeal resection in terms of specimen quality.

## Discussion

Decision-making in the management of bladder cancer relies upon the quality of tissue specimens and the precision of pathological diagnosis. TURBT is considered the gold standard for treating non-muscle invasive bladder cancer, allowing complete excision of tumors smaller than 1 cm during the procedure. ERBT has emerged as an advanced surgical approach, involving a circumferential incision in the bladder mucosa with a 5 mm safety margin from the lesion. This method facilitates removal of the entire tumor along with the underlying detrusor muscle, aligning with oncological surgical principles and permitting more accurate histopathological assessment, independent of the energy modality utilized [14–17].

The primary limitation of ERBT, as noted by most researchers, is tumor size, typically capping eligible lesions at 3 cm. Larger tumors generally require division into multiple fragments for extraction using devices such as retrieval bags, laparoscopic forceps, or morcellators—either to remove only the exophytic portion or the entire neoplasm [18]. However, recent evidence increasingly supports the efficacy of ERBT. Notably, a 2020 international consensus statement by Teoh et al., which incorporated two systematic reviews, a modified Delphi survey, and a consensus meeting, concluded with strong agreement (96%) regarding the evidence supporting ERBT. The publication underscored that ERBT is feasible even for bladder tumors exceeding 3 cm, and neither tumor count nor anatomical location pose intrinsic limitations [19].

Further, Dekel et al. recently reported on en bloc resection of large bladder tumors, highlighting that it may result in enhanced precision and operative control, potentially reducing complications such as bladder perforation. This technique remains consistent with key oncologic surgical principles, achieving negative resection margins in 94–99% of cases [11]. Additionally, ERBT markedly increases detection rates of detrusor muscle in pathology, which is important for oncological outcomes and therapeutic decision-making [20]. Recent meta-analysis by Li et al. published at 2026 in general support previous findings. In terms of pathology, ERBT resulted in higher detrusor muscle rate even in large tumor and lower residual tumor rate, but was not superior in mucosal muscle detection rate. Also some clinical advantages were noted (lower recurrence at 12 mo, but not at 36 mo, lower bladder perforation rate). Unfortunately, the authors did not assess morcellation value [21]. Despite these advances, widespread adoption of ERBT remains limited, and piecemeal TURBT continues to be the preferred surgical intervention for larger tumors amenable to fragmentary removal [22].

One of the steps that can promote ERBT with morcellation is to assess from a pathological point of view whether morcellation is a reasonable and adequate technique for pathological examination that does not compromise specimen quality and whether the surgeon needs to provide additional tissue from horizontal and vertical margins. If these goals are met, the method can appear in guidelines issued by pathology and urology societies, allowing its integration into educational programs and pathology reports. The Delphi method is applied in histopathology to determine best practices for diagnostic procedures, and a consensus level of 75% is typically considered sufficient.

In the current study, the two-round survey yielded a consensus on 8 out of 15 items addressing main issues in tumor grading and staging, and one item addressing morcellation as a method compared to standard piecemeal, not reaching the assigned agreement on the notion that it might be better. While an additional biopsy to assess vertical and horizontal margins is the most crucial aspect for accurate pathological examination and analysis, a consensus was not reached on this matter. Summing up, morcellation provides specimens of good quality that are suitable for comprehensive pathological assessment. Thus this method may be considered a feasible option in selected cases at the surgeon’s discretion. It should be highlighted, that 12/15 items were related solely to morcellated tissue, and pathologists answered them based on the digital scans. This was done because morcellation is not common and some pathologist might not have an extensive experience with such samples. The other one item was about comparison of morcellated specimen to intact lesion after ERBT, and two items were about tumors after morcellation vs. piecemeal resection. We did not provide pathologists with scans of such samples, because all of them had experience with such specimen and thus could make a comparison. However, a scan might be perceived in a different way than a sample under microscope, and quality of samples which pathologists handled before might differ, so our indirect comparison should be interpreted with caution considering these features. Our study is an expert consensus and not a study with direct comparison of the methods.

Three decades ago, morcellation was first employed to retrieve prostate tissue during enucleation, and Naspro et al. [23] demonstrated that it offered tissue sufficiently preserved for accurate pathological assessment and did not compromise prostate cancer detection. Morcellated tissue was also compared to specimens retrieved during open prostatectomy without significant differences in Gleason scores or prostate cancer staging [24]. 

It is well known that the detrusor is crucial for the precise staging of bladder cancer, as it determines the management strategy. Iscafe et al. [25] demonstrated on a small series of large tumors (> 3 cm) accurate diagnoses and staging in all cases and excellent quality of specimens after morcellation without fulguration artifacts. Another study conducted by Petov et al. comparing conventional TURBT to ERBT with morcellation in patients with large bladder tumors (> 3 cm) found more than 20% higher rates of detrusor muscle presence in the ERBT group, reaching almost 93% [26]. When discussing other possible findings in the bladder, one should mention a case report by Yuan et al., who managed to successfully resect a 5-cm rare inflammatory myofibroblastic tumor with malignant potential using en bloc and morcellation with subsequent proper immunohistochemistry staining [27]. Lastly, the WHO Classification and ICCR recommend sub-staging of pT1, but no indication is provided as to the methods to use for evaluating the extent of invasion. The anatomical approach to subclassification of pT1 tumors is based on the depth of invasion using muscularis mucosa as a histological landmark. T1 tumors can be divided into two subclasses (pT1a, b) [18]. A recent meta-analysis showed that clinicians should treat patients with T1b/c substages as having risk on a par with invasive bladder cancer [28]. Our panel of experts only reached a 60% agreement with the statement that morcellation allows for histological sub-staging. Moreover, this classification is not yet used in clinical settings.

A consensus among experienced pathologists is critical for standardizing pathological evaluation of morcellated specimens. In this consensus study, the goal was to address the narrative of compromised tissue quality when morcellation is used, despite the available evidence pointing to the contrary. Expert urologists generally adhere to standard practices and are skeptical of new techniques. The widespread use of ERBT for large tumors could result in pathologists gaining more experience in dealing with morcellated tissue and may provide better quality and accuracy when evaluating critical parameters of bladder tumors. This is of paramount importance for clinical management, as it would limit the number of cases where large tumors are labelled as muscle-invasive in advance and patients are subjected to radical surgeries. A review of the guidelines reveals that ERBT has started gaining the attention of the European Association of Urology but not the American Urological Association [29, 30]. Moreover, there is no mention of the morcellation technique in the management of large tumors. AUA guidelines label incomplete resection in large-volume high-grade tumors not amenable to complete endoscopic resection as it is unlikely to impact clinical management. Nevertheless, a large tumor does not necessarily imply muscle invasion and resecting it completely might prevent an unnecessarily invasive surgery for some selected patients, regardless of their suitability, according to performance status. ERBT with morcellation seems to be a convenient approach for such cases.

The study has a few limitations. A small cohort of experts (10 responders), although from different countries, may result in examinations that are non-comprehensive and lack statistical power. Besides, we enrolled only highly experienced pathologists in order to obtain the most reliable data, yet their conclusions may differ from their less experienced colleagues. Additionally, the study involved a low number of samples obtained by one experienced urologic practitioner after holmium laser ERBT, which may not be representative of how an average surgeon retrieves and handles the specimen. Optimal-case material together with high level of pathologists represents selection bias and limits generalizability of our study to the high-volume academic centers, while in small clinics quality of tissue assessment may be worse. Instead of microscopic slides, the pathologist were presented with their digital scans, that may influence perception. Finally, design of our study did not involve assessment of clinical outcomes, so our conclusions are limited to pathology.

## Conclusion

Tissue acquired after ERBT through morcellation permits dependable pathological evaluation, encompassing grading, staging, and identification of critical prognostic indicators such as carcinoma in situ, detrusor muscle, lymph vascular and perineural invasion. Morcellation is not perceived to compromise pathological evaluation based on expert consensus.

## Data Availability

No datasets were generated or analysed during the current study.
